# Development of a Validated Survey to Assess Virtual Interprofessional Education: Cross-Sectional Survey Study With Psychometric Validation

**DOI:** 10.2196/92933

**Published:** 2026-10-07

**Authors:** Erica Breyman, Dale Coffin, Bini John, Nancy Krisch, Kathleen Monahan, Xiaoyue Zhang, Erin Hulfish

**Affiliations:** 1 Renaissance School of Medicine at Stony Brook University, 100 Nicolls Rd, Stony Brook, NY, 11794, United States, 6313586139; 2 Occupational Therapy Program at Stony Brook University, Stony Brook, NY, United States; 3 Stony Brook University School of Nursing Department of Graduate Studies, Stony Brook, NY, United States; 4 Department of Physical Therapy at Stony Brook UniversityStony Brook, NY, United States; 5 Stony Brook School of Social Welfare, Stony Brook, NY, United States; 6 Biostatistical Consulting Core, Stony Brook, NY, United States

**Keywords:** telehealth, telemedicine, interprofessional education, assessment tools, digital health education

## Abstract

**Background:**

The COVID-19 pandemic led to the rapid integration of telemedicine into health care delivery. As a result, the ability to effectively collaborate in interprofessional virtual teams became a necessity, as did the development of educational opportunities designed to equip health care students with these skills. Currently, there is a lack of dedicated tools to assess learners’ ability and readiness to function as a well-coordinated virtual medical team.

**Objective:**

The primary objective of this study was to conduct a psychometric assessment and validation of measures evaluating graduate-level health professions learners’ experience and familiarity within virtual interprofessional settings. The secondary objective was to integrate these 2 validated subscales into a comprehensive survey tool for use in interprofessional education course evaluation, spanning domains including demographics, telehealth knowledge and skills, understanding interprofessional roles, communication, and teamwork.

**Methods:**

An interprofessional expert panel reviewed an initial item pool of 126 questions compiled from adaptations of previously validated instruments and newly developed items. Five experts with experience in interprofessional education and telehealth competencies independently rated each item, and the highest-ranked items were selected for distribution to graduate health professions students at Stony Brook University. The survey was administered during mandatory in-class instruction across the School of Medicine, School of Nursing, School of Health Professions, and School of Social Welfare between November 2023 and June 2024. Participation was voluntary, and responses were anonymous. Item reduction focused on the 33 experience items and 8 familiarity items using interitem correlations, the Cronbach α, item-to-total correlations, and exploratory factor analysis (EFA); confirmatory factor analysis was not conducted.

**Results:**

A total of 520 responses were obtained. After excluding records without any response to questions 9 or 10 and duplicate submissions, 393 records were included in the analysis. The final survey was reduced from 51 to 32 items, including 5 demographic items, 3 telehealth knowledge items, 17 retained experience items, and 5 retained familiarity items. For the experience domain, EFA identified 17 items loading onto 3 factors, accounting for 69.81%, 13.00%, and 9.58% of the variance. Internal consistency remained high, with the Cronbach α decreasing from 0.94 to 0.90. For the familiarity domain, EFA supported a refined 5-item structure, with the Cronbach α decreasing from 0.94 to 0.91.

**Conclusions:**

This EFA-based validation study provides preliminary psychometric support for 2 subscales assessing graduate health professions learners’ experience and familiarity within virtual interprofessional settings. Additional validation work is needed before broader claims can be made regarding the full survey’s ability to evaluate educational outcomes or clinical impact.

## Introduction

Prior to the COVID-19 pandemic, telemedicine use in the United States remained relatively limited because of reimbursement constraints, licensure barriers, workflow challenges, and insufficient training for both clinicians and learners [1]. The pandemic accelerated adoption across health systems, and telemedicine rapidly became embedded in routine care delivery, creating new expectations for health professions learners to communicate, assess, and collaborate effectively in technology-mediated environments [2,3]. As virtual care has persisted beyond the acute pandemic period, telehealth competence is now understood to include not only technical and communication skills but also the ability to function within distributed, interprofessional teams that coordinate care across professions, settings, and patient needs [2,3].

A growing body of recent scholarship has described virtual interprofessional education (IPE) initiatives in a range of settings, including virtual team visits, interprofessional telehealth curricula, and online learning activities designed to prepare students for collaborative virtual care [4-8]. These studies suggest that virtual IPE can support role clarification, communication, teamwork, and confidence with telehealth while also revealing persistent challenges related to workflow adaptation, learner preparedness, and unequal exposure to telehealth skills across disciplines [5-8]. Other recent reports have shown that telehealth-focused curricula can improve selected competencies and self-efficacy among health professions learners, but outcomes vary by educational design and context, and many evaluations rely on project-specific or locally developed measures rather than instruments tailored to virtual interprofessional practice [5-7]. In parallel, competency frameworks such as the Association of American Medical Colleges telehealth competencies and the updated Interprofessional Education Collaborative core competencies have underscored the importance of communication, teamwork, ethics, patient safety, and role clarity in virtual care; however, these frameworks do not themselves provide a validated survey specifically designed to assess learners’ experience and familiarity with functioning in virtual interprofessional teams [9,10].

To inform survey development, we conducted a literature review of existing validated IPE questionnaires and related tools assessing communication, team functioning, collaboration, and simulation-based learning [11-14]. This review identified several well-established instruments with overlapping domains relevant to interprofessional learning, including attitudes toward teamwork, collaboration, communication, and professional roles. However, the available instruments were developed largely for in-person IPE, general collaborative practice, or simulation settings rather than for the evaluation of education conducted in virtual care environments [11-13]. Virtual IPE requires learners to coordinate across distributed settings, communicate through technology-mediated encounters, establish role clarity without shared physical presence, anticipate workflow needs in remote team-based care, and apply telehealth-specific communication strategies. These competencies differ from traditional in-person IPE because the educational and clinical interactions occur through digital platforms, require explicit attention to virtual etiquette and communication tools, and depend on learners’ familiarity with virtual care workflows. Recent literature similarly highlights the expansion of virtual IPE and telehealth education programs but offers limited evidence on standardized, psychometrically tested measures that capture learners’ prior experience with virtual teamwork and their familiarity with performing in telehealth-enabled interprofessional settings [11-14]. Thus, an important knowledge gap remains: educators increasingly need rigorous tools to evaluate virtual IPE across diverse learner groups, yet there is no widely validated instrument specifically designed for this purpose. The primary objective of this study was to conduct a psychometric assessment and validation of measures evaluating graduate-level health professions learners’ experience and familiarity within virtual interprofessional settings. The secondary objective was to integrate these two validated subscales into a comprehensive survey tool for use in IPE course evaluation, spanning domains including demographics, telehealth knowledge and skills, understanding interprofessional roles, communication, and teamwork.

## Methods

### Survey Development

A literature search was conducted to review existing validated IPE questionnaires [11-14]. Although these surveys had notable and overlapping IPE domains, including communication, team functioning, and collaboration, they were not designed specifically for the evaluation of education in virtual environments. As such, the authors generated the initial item pool via question development and modification of existing validated questionnaires, including questions from the Interprofessional Socialization and Valuing Scale 9A, the Brief Attitudes Survey for Interprofessional Collaborative Learning, the Attitudes Toward Health Care Teams Scale, the Team Skills Scale, the Interprofessional Collaboration Scale, and the Simulation Effectiveness Tool–Modified (Multimedia Appendix 1) [11-14].

### Creation of the Initial Survey Database and Expert Panel Review

A total of 126 questions were compiled into the initial database, covering five key domains: (1) demographics, (2) telehealth knowledge and skills, (3) understanding interprofessional roles, (4) communication, and (5) teamwork. A core interprofessional group of 5 experts was recruited to refine the initial 126-item survey into a 50-item survey. These individuals were members of the Interprofessional Education Committee at Stony Brook University, convened to guide the development of future IPE courses. This panel included representatives from the School of Medicine, School of Nursing, School of Health Professions, and School of Social Welfare with extensive knowledge in both IPE and telehealth core competencies [9,10]. These representatives individually assigned a ranking to each item. Individual scores were summed and divided by the number of rankings assigned to each item, and the top 50 questions were chosen for widespread distribution. These were grouped into the following sets of questions: demographic-related information (questions 1-8), questions of experience in interprofessional teams (question 9), and questions of familiarity in interprofessional teams (question 10). Demographic items collected information on participants’ school; sex; ethnicity; race; age; and perceived knowledge, skill, and usability of telehealth. Originally, question 9 contained 33 items, and question 10 contained 8 items. Psychometric validation was performed exclusively for questions 9 and 10, and each section was analyzed independently.

### Survey Dissemination

A convenience sampling approach was used targeting graduate health professions students participating in required coursework across the School of Medicine, School of Nursing, School of Health Professions, and School of Social Welfare at Stony Brook University. Recruitment and survey distribution took place between November 2023 and June 2024 during mandatory in-class instruction associated with IPE coursework. Students were invited to complete the survey electronically during class sessions. Participation was voluntary, responses were anonymous, instructors did not have access to individual responses, no grades or evaluations were linked to participation, and no incentives were provided. A total of 520 students responded. Records were excluded if they had no response to either question 9 or question 10 (n=122), leaving 398 records. Five duplicate submissions were identified and removed, resulting in 393 records for analysis. No separate 95% completion threshold was applied.

### Data Analysis

Analyses followed standard psychometric validation procedures for scale development and exploratory factor analysis (EFA) [15-19]. Differences in the distribution of participants’ responses across the 4 schools were evaluated using chi-square tests, with exact *P* values generated via Monte Carlo simulation to account for small cell counts and potential sparsity in the data. The item reduction analysis focused on refinement of the 2 conceptually distinct domains: experience in interprofessional teams (question 9) and familiarity in interprofessional teams (question 10). These domains were analyzed separately a priori because experience and familiarity represent related but distinct constructs. Descriptive analyses were first conducted to examine relationships among items in questions 9 and 10 using polychoric correlation heat maps to visualize estimated correlations between ordinal items and confirm the expected separation of the 2 domains. Instrument reliability for questions 9 and 10 was evaluated using the Cronbach α, with values between 0.7 and 0.9 considered acceptable and values above 0.9 considered indicative of potential item redundancy [15-17]. Item-to-total correlations were used as an initial item performance screen to identify items with limited association with their intended domain; items with item-to-total correlations of less than 0.3 were removed before EFA. This screening step was used to reduce poorly performing items while recognizing that EFA was subsequently used to evaluate multidimensional factor structure within each domain. EFA using principal factor extraction with oblique Promax rotation was performed. Suitability for factor analysis was confirmed via the Kaiser-Meyer-Olkin (KMO) measure (threshold>0.5) [16,17]. Parallel analysis determined the number of factors to retain by comparing observed eigenvalues with those obtained from simulated data. Items were retained if they demonstrated a primary factor loading of at least 0.50, did not demonstrate substantial cross-loading, and had a difference of at least 0.20 between the primary and next-highest loading. Items that did not meet these criteria were removed to produce a clearer, more interpretable factor structure [18,19].

All statistical procedures were performed in SAS (version 9.4; SAS Institute Inc).

### Ethical Considerations

This study was submitted to the Stony Brook University Institutional Review Board (IRB) for preliminary review and was determined not to constitute human subject research requiring further IRB review or approval (IRB2023-00080). Participation in the survey was voluntary. Completion of the anonymous survey was considered implied consent; no written informed consent was obtained because no identifiable private information was collected and the activity was determined not to require further IRB review. Privacy and confidentiality were protected by collecting responses anonymously, storing data in deidentified aggregate form for analysis, and ensuring that instructors did not have access to individual responses. No grades, evaluations, or other academic consequences were linked to participation or nonparticipation. Participants received no compensation or incentives for completing the survey.

## Results

### Overview

A total of 520 responses were received (Figure 1). After excluding records with no response to either question 9 or 10 and removing duplicate submissions, 393 records were included in the analysis, with the distribution of participant characteristics summarized in Table 1.

x`Question 9, which assessed experience in interprofessional teams, consisted of 33 items, and question 10, which assessed familiarity in interprofessional teams, consisted of 8 items. Questions 9 and 10 were analyzed separately because experience and familiarity were defined a priori as conceptually distinct constructs. A polychoric correlation heat map was used to examine correlations among items and confirmed the expected separation of questions 9 and 10 into distinct blocks (Figure 2). Item reduction was therefore conducted separately for questions 9 and 10.

**Figure 1.**
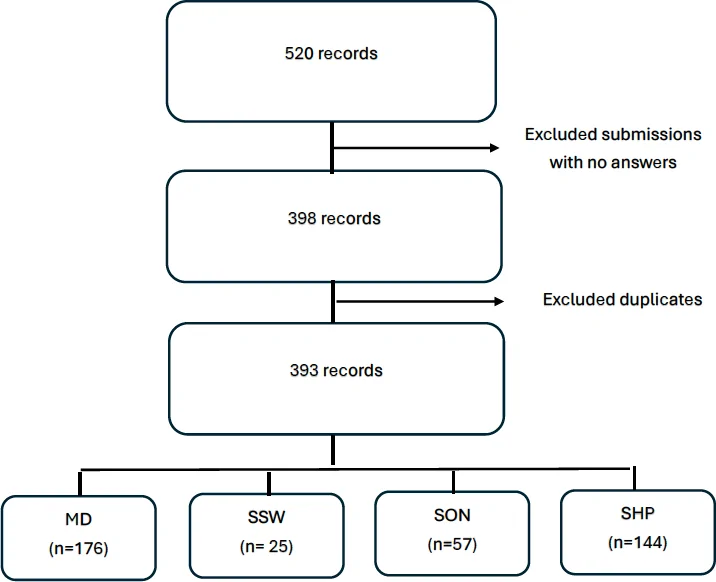
Flow diagram of records included in this cross-sectional survey study with psychometric validation of a virtual interprofessional education instrument among graduate health professions students at Stony Brook University, Stony Brook, New York, United States. Surveys were administered during mandatory in-class interprofessional education instruction across the School of Medicine (MD), School of Nursing (SON), School of Health Professions (SHP), and School of Social Welfare (SSW) from November 2023 to June 2024; the figure shows total responses, exclusions for missing question 9 and 10 data, duplicate removal, and the final analytic sample.

**Table 1.** Demographic characteristics and prior telehealth experience of participants in a cross-sectional survey study with psychometric validation of a virtual interprofessional education instrument among graduate health professions students at Stony Brook University, Stony Brook, New York, United States. The analytic sample included 393 students from the School of Medicine (MD), School of Nursing (SON), School of Health Professions (SHP), and School of Social Welfare (SSW) who completed relevant survey items during mandatory in-class interprofessional education instruction from November 2023 to June 2024 (N=393).

Item and level	Total, n (%)	MD (n=167), n (%)	SSW (n=25), n (%)	SHP (n=144), n (%)	SON (n=57), n (%)
Age (y)					
≤21	4 (1.3)	3 (1.8)	0 (0)	1 (0.7)	0 (0)
22-27	296 (75.3)	153 (91.6)	14 (56)	124 (86.1)	5 (8.8)
28-33	40 (10.2)	9 (5.4)	5 (20)	11 (7.6)	15 (26.3)
34-39	20 (5.1)	1 (0.6)	0 (0)	3 (2.1)	16 (28.1)
40-45	8 (2.0)	1 (0.6)	2 (8)	2 (1.4)	3 (5.3)
46-51	13 (3.3)	0 (0)	4 (16)	0 (0)	9 (15.8)
52-57	9 (2.3)	0 (0)	0 (0)	1 (0.7)	8 (14.0)
58-63	2 (0.5)	0 (0)	0 (0)	1 (0.7)	1 (1.8)
≥64	1 (0.3)	0 (0)	0 (0)	1 (0.7)	0 (0)
Prior telehealth experience					
None	115 (29.3)	68 (40.7)	2 (8)	38 (26.4)	7 (12.3)
Professional occasional use	83 (21.1)	39 (23.4)	6 (24)	24 (16.7)	14 (24.6)
Professional moderate use	50 (12.7)	18 (10.8)	4 (16)	22 (15.3)	6 (10.5)
Professional extensive use	14 (3.6)	7 (4.2)	3 (12)	2 (1.4)	2 (3.5)
Personal use	131 (33.3)	35 (21.0)	10 (40)	58 (40.3)	28 (49.1)

**Figure 2.**
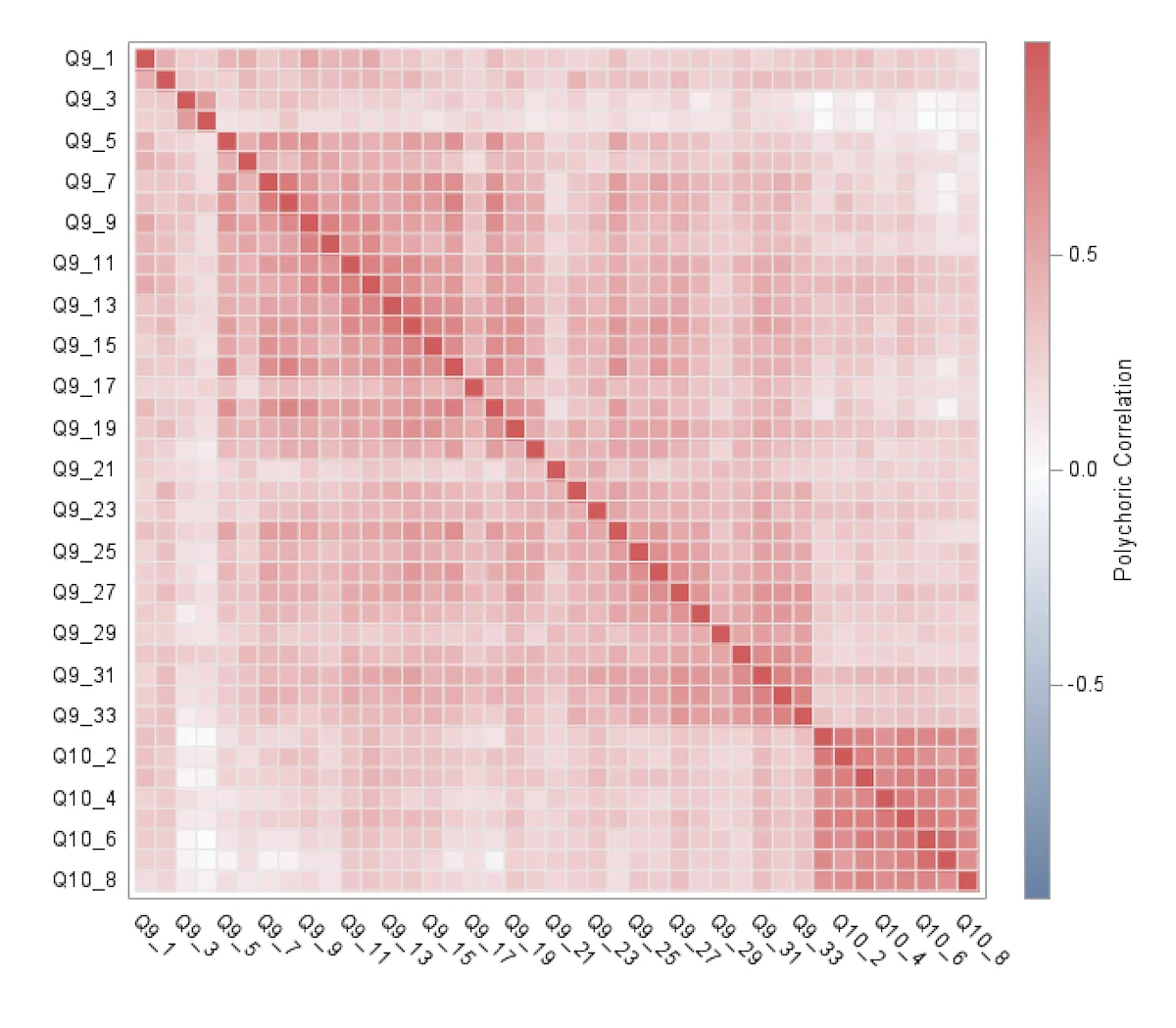
Polychoric correlation heat map from a cross-sectional psychometric validation study of a virtual interprofessional education survey among graduate health professions students at Stony Brook University, Stony Brook, New York, United States, administered from November 2023 to June 2024. The heat map displays item-level correlations for question 9, assessing experience in interprofessional teams, and question 10, assessing familiarity in interprofessional teams, and supports the a priori decision to analyze these 2 domains separately because the items clustered into distinct correlation blocks.

### Question 9: Experience in Interprofessional Teams

The original 33-item section demonstrated strong internal consistency, with a Cronbach α of 0.94. Four items with an item-to-total correlation below 0.3 were removed. Using the remaining 29 items, the data showed high interitem correlations, as indicated by a KMO score of 0.94, confirming its suitability for the questions to move onto the next stage of evaluation for factor analyses. EFA identified 3 meaningful factors that explained 69.81%, 13.00%, and 9.58% of the variance. A total of 6, 8, and 3 items loaded clearly onto the 3 factors, resulting in a refined 17-item version.

The internal consistency of the refined scale remained strong, with an overall Cronbach α of 0.9 and factor-specific Cronbach α values of 0.88, 0.89, and 0.77. Internal consistency reliability was also assessed within each school using the reduced 17-item version, and acceptable reliability was confirmed both overall and within each factor (Table 2).

**Table 2.** Exploratory factor analysis results for question 9, the experience in interprofessional teams subscale, in a cross-sectional psychometric validation study of a virtual interprofessional education survey among graduate health professions students at Stony Brook University, Stony Brook, New York, United States. Survey data were collected during mandatory in-class interprofessional education instruction from November 2023 to June 2024; the table presents retained items from the original 33-item question 9 section and their factor loadings after item reduction.

Item	Label	Factor loadings		
		Factor 1	Factor 2	Factor 3
Factor 1				
Q9_8	During their education, health professional students should be involved in teamwork with students from different disciplines in order to understand their respective roles.	0.83^a^	−0.04	−0.07
Q9_16	Learning from students from other professions will make me a more effective member of an interprofessional team.	0.82	0.07	−0.12
Q9_18	The team approach improves the quality of care to patients.	0.77	−0.04	−0.03
Q9_7	Health professional students from different disciplines should be educated on establishing virtual collaborative relationships with one another.	0.75	0.04	−0.08
Q9_9	I will be able to share and exchange ideas in a team discussion.	0.67	−0.19	0.34
Q9_5	Working with students from different disciplines enhances my education.	0.66	−0.09	0.08
Factor 2				
Q9_25	The virtual team approach makes the delivery of care more efficient.	0.07	0.77	−0.12
Q9_32	Team members cooperate with the way care is organized.	0.02	0.74	0.03
Q9_31	Team members are willing to discuss individuals’ issues virtually.	0.01	0.70	0.08
Q9_33	Team members anticipate when they will need others’ help in virtual settings.	−0.09	0.70	0.14
Q9_27	The team has a good understanding about their respective responsibilities in virtual settings.	0.08	0.70	0.09
Q9_28	Team members are usually willing to take into account the convenience of individuals when planning their work.	−0.05	0.67	0.06
Q9_30	Individuals on the team share similar ideas about how to treat patients.	−0.04	0.59	0.11
Q9_22	Developing a virtual patient care plan with other team members avoids errors in delivering care.	0.07	0.51	0.07
Factor 3				
Q9_4	I am familiar with different formalized communication tools utilized within interprofessional Telehealth teams.	−0.26	0.22	0.71
Q9_3	I have an understanding of how to use Motivational Interviewing to enhance my interview skills with clients/patients.	−0.08	0.08	0.65
Q9_2	I feel that I can communicate effectively using Telehealth.	0.03	0.18	0.57

a Italics indicate the primary loading in the final 17-item, 3-factor structure.

### Question 10: Familiarity in Interprofessional Teams

The original 8-item section demonstrated high internal consistency, with a Cronbach α of 0.94. All items showed item-to-total correlations above 0.30, making them all considered in factor analysis. A KMO score of 0.92 further confirmed strong correlations among the items (Table 3).

**Table 3.** Exploratory factor analysis (EFA) results for question 10, the familiarity in interprofessional teams subscale, in a cross-sectional psychometric validation study of a virtual interprofessional education survey among graduate health professions students at Stony Brook University, Stony Brook, New York, United States. Survey data were collected during mandatory in-class interprofessional education instruction from November 2023 to June 2024; the table compares 1- and 2-factor EFA solutions for the original 8-item question 10 section and identifies the retained 5-item structure.

Item	Label	1-factor EFA—factor loadings	2-factor EFA	
		Factor loadings	Factor 1 loadings	Factor 2 loadings
Q10_6	Address clinical issues succinctly in virtual interdisciplinary meetings.	0.87	0.88^a^	0.09
Q10_7	Develop a virtual interdisciplinary care plan.	0.79	0.86	0.01
Q10_5	Strengthen cooperation among disciplines virtually.	0.88	0.47	0.49
Q10_4	Handle disagreements effectively in virtual settings.	0.79	0.44	0.42
Q10_8	Recognize when the team is not functioning well in a virtual environment.	0.77	0.42	0.42
Q10_3	Identify contributions to patient care that different disciplines can offer.	0.84	0.36	0.55
Q10_1	Function effectively in a virtual interdisciplinary team.	0.80	0.30	0.58
Q10_2	Treat team members as colleagues in virtual settings.	0.77	-–0.07	0.95

Parallel analysis initially indicated a single-factor solution, suggesting that all 8 items could be represented by 1 factor. However, for the purpose of item reduction, a 2-factor model was explored, and this model converged successfully. One factor contained 3 items, and the other factor contained 2 items. In the unrotated solution, factors 1 and 2 explained 93.03% and 6.98% of the variance, respectively. The simplified 5-item version maintained strong reliability, with a Cronbach α of 0.91 for the overall scale and of 0.90 and 0.88 for the 2 factors. The reduced instrument showed consistent reliability across various professional program groups, although small sample sizes from the School of Social Welfare and the School of Nursing may have slightly influenced the results.

### Final Survey

To ensure the comprehensive evaluation of IPE in virtual environments, the final survey compilation incorporated demographic factors to enhance its applicability across diverse settings. These factors were carefully integrated to capture variations in responses attributable to participants’ backgrounds, such as age, professional discipline, and educational level and led to the final survey total questions to 32 items from the initial 51 questions (Multimedia Appendix 2). Multimedia Appendix 3 identifies each candidate item, whether it was retained in the final instrument or removed during refinement, whether it was adapted from an existing validated interprofessional or teamwork instrument vs newly developed for this study, and whether the item captures a virtual-specific competency.

## Discussion

### Principal Findings

This study aimed to develop and conduct preliminary psychometric validation of survey subscales assessing IPE conducted in a virtual environment. The survey development process included evaluation and thematic grouping of items adapted from previously validated IPE tools, ranking and review by an expert panel with expertise in IPE and telehealth competencies, and distribution to graduate health professions students across multiple schools. The psychometric analysis focused on 2 sections: experience in interprofessional teams (question 9) and familiarity in interprofessional teams (question 10). EFA supported refinement of question 9 from 33 items to a 17-item, 3-factor structure and refinement of question 10 from 8 items to a 5-item structure. Confirmatory factor analysis was not conducted; therefore, the findings should be interpreted as preliminary evidence supporting the internal structure and reliability of these 2 subscales.

Prior studies have described virtual and telehealth-based IPE curricula that target communication, teamwork, role clarity, learner confidence, and telehealth readiness [4-7]. The present study did not directly evaluate whether learners improved in these outcomes. Rather, it contributes to this literature by developing and preliminarily validating two survey subscales intended to measure learners’ reported experience and familiarity within virtual interprofessional settings. This distinction is important because validated measurement tools are needed before educational programs can be compared reliably across contexts or linked to learner outcomes.

The present study provides preliminary psychometric support for two subscales designed to assess learners’ experience and familiarity within virtual interprofessional environments. Several validated instruments exist to assess interprofessional attitudes, teamwork, collaboration, and simulation-based learning; however, many were developed primarily for in-person or simulation-based settings rather than for distributed, technology-mediated care [11-13]. The retained items include content related to virtual collaboration, telehealth communication, virtual care planning, and functioning within virtual interdisciplinary teams. However, because only the experience and familiarity subscales were evaluated using EFA, the findings should not be interpreted as validation of the entire survey or as definitive evidence that all virtual-specific competencies have been fully captured. Further studies are needed to confirm the factor structure, examine construct validity, and evaluate relationships with educational outcomes.

### Limitations

This study has several limitations. First, uneven participation across professional groups, particularly lower representation from the School of Nursing and School of Social Welfare, may have influenced reliability estimates and limited generalizability. Second, data were collected from a single academic medical center, which may further restrict broader applicability. In addition, the reliance on self-reported data introduces the potential for response and social desirability bias. The cross-sectional design precludes assessment of test-retest reliability and limits evaluation of the temporal stability of the instrument. Finally, as all data were obtained from a single survey source at a single time point, there is potential for common method variance, which may have inflated observed associations among items.

Despite these limitations, the stepwise development process and exploratory psychometric evaluation provide preliminary support for the reliability and internal structure of the two validated subscales.

### Conclusions

The Virtual Interprofessional Education Survey includes two subscales with preliminary EFA-based psychometric support for assessing graduate health professions learners’ experience and familiarity within virtual interprofessional settings. The retained items address aspects of virtual teamwork, telehealth communication, interdisciplinary collaboration, and virtual care planning. These findings support continued refinement and evaluation of the instrument, including confirmatory factor analysis, assessment of construct validity, and testing in broader learner populations, before the tool is used to make stronger claims about educational outcomes or clinical impact.

## Supplementary material

10.2196/92933 Multimedia Appendix 1 Ranking of the generated initial item pool.

10.2196/92933 Multimedia Appendix 2 Final virtual interprofessional education survey.

10.2196/92933 Multimedia Appendix 3 Full 126 initial telehealth and interprofessional competencies with outcomes of retention, adaptation, or removal.

## Abbreviations

EFA: exploratory factor analysis
IPE: interprofessional education
IRB: Institutional Review Board
KMO: Kaiser-Meyer-Olkin
