# Supplementary material for: Development of a Validated Survey to Assess Virtual Interprofessional Education: Cross-Sectional Survey Study With Psychometric Validation
Source: JMIR Form Res. 2026 Oct 7;10:e92933. doi: 10.2196/92933 (PMC13645316; doi:10.2196/92933)
Supplement: Supplementary file 1 [file formative-v10-e92933-s001.docx]

Appendix A:

| **Questions** | **Reviewer**  **1** | **Reviewer 2** | **Reviewer 3** | **Reviewer 4** | **Reviewer 5** |
| --- | --- | --- | --- | --- | --- |
| Demographic Information | | | | | |
| Professional Program |  |  | 1 |  |  |
| Gender |  |  | 1 |  |  |
| Primary Ethnicity |  |  |  |  |  |
| Primary Race |  |  |  |  |  |
| Age |  |  | 1 |  |  |
| Please indicate the choice below that best describes the amount of experience you have had with using telemedicine/telehealth prior to the course:  None  Professional occasional use  Professional moderate use  Professional extensive use  Personal use |  |  |  |  |  |
| On a scale of 0-5 (with 0 being least familiar and 5 being most familiar), please Rate Your Level of Understanding of: |  |  |  |  |  |
| Medical Students and their Profession PRIOR to the start of the class |  |  | 1 |  |  |
| the Social Welfare Students and their Profession PRIOR to the start of class |  |  | 1 |  |  |
| the Health Professional Students and their Profession PRIOR to the start of class |  |  | 1 |  |  |
| the Nursing Students and their Profession PRIOR to the start of class |  |  | 1 |  |  |
| Please rate yourself on each of the following knowledge and skills regarding Telehealth using a five-point scale where (5 indicates excellent and 1 indicates poor) |  |  |  |  |  |
| Knowledge of Telehealth etiquette | 3 | 2 | 1 |  | 2 |
| Confidence in using proper patient/provider communication skills in a Telehealth setting | 2 | 1 | 2 |  | 1 |
| Knowledge of common uses of Telemedicine in my profession | 1 | 3 | 3 |  | 3 |
| Virtual Interprofessional Questions | | | | | |
| Please respond to each question using a five-point scale from Strongly Agree to Strongly Disagree. Choose the one number that you feel best fits your experience. |  |  |  |  |  |
| I feel that I can communicate effectively within inter-professional teams |  | 1 | 1 |  | 1 |
| I feel that I can communicate effectively using Telehealth | 1 | 2 | 3 |  | 2 |
| I have an understanding of how to use Motivational Interviewing to enhance my interview skills with clients/patients |  | 4 | 4 |  | 3 |
| I am familiar with different formalized communication tools utilized within interprofessional Telehealth teams |  | 3 | 2 |  | 4 |
| Please respond to each question using a five-point scale from Strongly Agree to Strongly Disagree. |  |  |  |  |  |
| Working with students from different disciplines enhances my education | 2 |  | 1 |  | 9 |
| My role within an inter-professional team is clearly defined | 1 | 1 | 2 |  | 8 |
| Patient/client satisfaction is improved when care is delivered by an interprofessional team | 9 | 7 | 3 |  | 5 |
| Participating in educational experiences with students from different disciplines enhances my ability to work on an interprofessional team | 3 | 6 | 9 |  | 2 |
| I have an understanding of the courses taken by, and training requirements of, other health professionals | 8 | 8 | 8 |  | 6 |
| Health professional students from different disciplines should be educated on establishing virtual collaborative relationships with one another | 4 | 2 | 4 |  | 3 |
| I understand the roles of other health professionals within an interprofessional team | 6 | 3 | 5 |  | 7 |
| Patient/client-centeredness increases when care is delivered by a virtual interprofessional team | 5 | 5 | 6 |  | 4 |
| During their education, health professional students should be involved in teamwork with students from different disciplines in order to understand their respective roles | 7 | 4 | 7 |  | 1 |
| Please respond to each question using a five-point scale from Strongly Agree to Strongly Disagree. Choose the answer that you feel best fits your experience. Please indicate the degree to which you currently hold or display each of the beliefs, behaviors and attitudes that are described. Please respond to these questions based on your expectations of each question prior to the Interprofessional Telehealth course. |  |  |  |  |  |
| I will be able to share and exchange ideas in a team discussion. | 6 | 9 | 1 | 1 | 2 |
| I will gain an enhanced perception of myself as someone who engages in inter-professional practice. | 5 | 8 | 6 | 6 | 4 |
| I will be comfortable speaking out within the team when others are not keeping the best interests of the client in mind. | 7 | 7 | 7 | 7 | 8 |
| I believe that the best decisions are made when members openly share their views and ideas. | 8 | 6 | 8 | 10 | 3 |
| I will feel comfortable in describing my professional role to another team member in a virtual environment | 1 | 5 | 9 | 3 | 7 |
| I will gain an enhanced awareness of roles of other professionals on a team in a virtual environment | 2 | 1 | 2 | 2 | 6 |
| I will gain an appreciation for the importance of having the client and family as members of the team in a virtual environment | 9 | 2 | 3 | 5 | 9 |
| I will be comfortable engaging in shared decision making with the clients as part of the IP team in a virtual environment | 3 | 3 | 4 | 4 | 1 |
| I will be comfortable and accepting responsibility delegated to me within a team in a virtual environment | 4 | 4 | 5 | 8 | 5 |
| Please respond to each question using a five-point scale from Strongly Agree to Strongly Disagree. Choose the answer that you feel best fits your experience. Please indicate the degree to which you currently hold or display each of the beliefs, behaviors and attitudes that are described. |  |  |  |  |  |
| Patients would ultimately benefit if Interprofessional student teams worked together in a virtual environment to address household concerns | 9 |  | 1 |  |  |
| Collaborative learning with students with students will increase my ability to positively impact the patient's household | 7 | 9 | 10 | 5 | 9 |
| Collaborative learning will help me understand my own professional limitations | 8 | 7 | 2 | 4 | 2 |
| Collaborative learning will help me understand the value of other health professionals | 6 | 6 | 3 | 3 | 5 |
| Collaborative learning with other students from other professions will help me to communicate better with patients | 5 | 1 | 4 | 6 | 8 |
| Collaborative learning with other students from other professions will help me to communicate better with other professionals | 4 | 5 | 5 | 7 | 7 |
| Collaborative learning during Telehealth visits is likely to improve services for patients and their households | 3 | 2 | 6 | 2 | 1 |
| I would welcome the opportunity to participate in lectures, tutorials, or workshops with students from other professions | 10 | 8 | 7 | 8 | 4 |
| Learning from students from other professions will make me a more effective member of an Interprofessional team | 1 | 3 | 8 | 1 | 3 |
| Learning with students from other professions would further develop my teamwork skills | 2 | 4 | 9 | 9 | 6 |
| "IN MY OPINION" (six-point scale from Strongly Agree to Strongly Disagree): |  |  |  |  |  |
| Working in teams unnecessarily complicates things most of the time | 1 |  | 10 |  | 17 |
| The team approach improves the quality of care to patients | 15 | 4 | 1 | 1 | 2 |
| Virtual team meetings foster communication among team members from different disciplines | 13 | 3 | 11 | 8 | 14 |
| Physicians have the right to alter patient care plans developed by the team | 11 |  | 12 |  | 13 |
| Patients receiving team care are more likely than other patients to be treated as whole persons | 4 | 5 | 2 | 7 | 1 |
| A team's primary purpose is to assist physicians in achieving treatment goals for patients | 6 | 13 | 13 |  | 16 |
| Working on a team keeps most health professionals enthusiastic and interested in their jobs | 19 | 14 | 3 | 6 | 12 |
| Patients are less satisfied with their care when it is provided by a virtual team | 7 | 6 | 14 | 5 | 15 |
| Developing a patient care plan with other team members avoids errors in delivering care | 17 | 7 | 4 | 4 | 3 |
| When developing interdisciplinary patient care plans, much time is wasted translating jargon from other disciplines | 10 | 12 | 15 |  | 11 |
| Health professionals working on virtual teams are more responsive than others to the emotional and financial needs of patients | 21 | 11 | 16 | 2 | 10 |
| Developing a virtual interdisciplinary patient care plan is excessively time consuming | 2 |  | 17 | 10 | 9 |
| The physician should not always have the final word in decisions made by health care teams | 18 | 8 | 9 |  | 20 |
| The give and take among team members help them make better patient care decisions | 3 | 10 | 5 |  | 4 |
| In most instances, the time required for team meetings could be better spent in other ways | 16 |  | 18 |  | 8 |
| The physician has the ultimate legal responsibility for decisions made by the team | 5 | 15 | 19 |  | 21 |
| Hospital patients who receive team care are better prepared for discharge than other patients | 14 | 7 | 20 | 9 | 5 |
| Physicians are natural team leaders | 8 |  | 21 |  | 19 |
| The virtual team approach makes the delivery of care more efficient | 9 | 1 | 22 |  | 6 |
| The virtual team approach permits health professionals to meet the needs of family caregivers as well as patients | 12 | 2 | 7 |  | 7 |
| Having to report observations to the team helps team members better understand the work of other health professionals | 20 | 9 | 8 |  | 18 |
| Please rate your ability to carry out each of the following tasks (5-point scale from Poor to Excellent) |  |  |  |  |  |
| Function effectively in a virtual interdisciplinary team | 14 | 3 | 10 | 1 | 1 |
| Treat team members as colleagues | 6 | 2 | 11 |  | 3 |
| Identify contributions to patient care that different disciplines can offer | 10 | 6 | 1 | 7 | 4 |
| Apply your knowledge of geriatric principles for the care of older persons in a team care setting | 17 | 14 | 16 |  | 17 |
| Ensure that patient/family preferences/goals are considered when developing the team's care plan | 16 | 13 | 2 | 6 | 5 |
| Handle disagreements effectively | 7 | 16 | 3 | 5 | 6 |
| Strengthen cooperation among disciplines | 5 | 4 | 4 |  | 7 |
| Carry out responsibilities specific to your discipline's role on a team | 15 | 5 | 5 |  | 8 |
| Address clinical issues succinctly in virtual interdisciplinary meetings | 1 | 15 | 6 |  | 9 |
| Participate actively in virtual team meetings | 4 | 12 | 7 |  | 16 |
| Develop an interdisciplinary care plan | 8 | 1 | 8 |  | 12 |
| Adjust your care to support the team goals | 13 | 7 | 9 |  | 10 |
| Develop intervention strategies that help patients attain goals | 12 | 8 | 12 |  | 11 |
| Raise appropriate issues in virtual team meetings | 2 | 11 | 13 |  | 12 |
| Recognize when the team is not functioning well | 3 | 9 | 1 | 2 | 14 |
| Intervene effectively to improve team functioning | 9 | 10 | 14 | 3 | 15 |
| Help draw out team members who are not participating actively in meetings | 11 | 17 | 15 | 4 | 16 |
| Four-point scale from Strongly Agree to Strongly Disagree: |  |  |  |  |  |
| The team has a good understanding about their respective responsibilities | 8 | 3 | 1 |  | 12 |
| Team members are usually willing to take into account the convenience of individuals when planning their work | 7 | 2 | 5 |  | 11 |
| I feel that patient treatment and care are not adequately discussed between and among team members | 6 | 1 | 7 |  | 10 |
| Individuals on the team share similar ideas about how to treat patients | 9 | 9 | 8 |  | 1 |
| Team members are willing to discuss individuals’ issues | 10 | 6 | 2 |  | 5 |
| Team members cooperate with the way care is organized | 11 | 5 | 3 |  | 4 |
| Team members would be willing to cooperate with new, agreed upon practices | 12 | 4 | 6 |  | 6 |
| Individuals are not usually asked for their opinions | 5 | 7 | 9 |  | 9 |
| Team members anticipate when they will need others’ help | 4 | 8 | 4 |  | 2 |
| Important information is always passed between and among team members | 13 | 10 | 5 |  | 3 |
| Disagreements within the team often remain unresolved | 2 | 13 | 10 |  | 13 |
| Some individuals think their work is more important than the work of others on the team | 1 | 11 | 11 |  | 8 |
| Some individuals would not be willing to discuss new practices with other team members | 3 | 12 | 12 |  | 7 |
| After completing a simulated clinical experience, please respond to the following statements by picking your response. (Strongly Agree (1), Somewhat Agree (2), Do Not Agree (3)) |  |  |  |  |  |
| Prebriefing increased my confidence |  | 16 | 1 |  | 18 |
| Prebriefing was beneficial to my learning |  | 17 | 2 |  | 14 |
| I am better prepared to respond to changes in my patient’s condition |  | 18 | 3 |  | 4 |
| I developed a better understanding of the pathophysiology |  | 2 | 19 |  | 5 |
| I am more confident of my assessment skills |  | 14 | 4 |  | 1 |
| I felt empowered to make clinical decisions |  | 12 | 5 |  | 2 |
| I developed a better understanding of medications. (Leave blank if no medications in scenario) |  | 13 | 20 |  | 6 |
| I had the opportunity to practice my clinical decision making skills. |  | 5 | 6 |  | 3 |
| I am more confident in my ability to prioritize care and interventions. |  | 4 | 7 |  | 7 |
| I am more confident in communicating with my patient. |  | 11 | 8 |  | 12 |
| I am more confident in my ability to teach patients about their illness and interventions |  | 10 | 9 |  | 13 |
| I am more confident in my ability to report information to health care team |  | 3 | 10 |  | 7 |
| I am more confident in providing interventions that foster patient safety |  | 15 | 11 |  | 8 |
| I am more confident in using evidence-based practice to provide care |  | 1 | 12 |  | 9 |
| Debriefing contributed to my learning |  | 6 | 13 |  | 15 |
| Debriefing allowed me to verbalize my feelings before focusing on the scenario |  | 7 | 14 |  | 16 |
| Debriefing was valuable in helping me improve my clinical judgment |  | 8 | 15 |  | 18 |
| Debriefing provided opportunities to self-reflect on my performance during simulation |  | 9 | 16 |  | 17 |
| Debriefing was a constructive evaluation of the simulation |  |  | 17 |  | 11 |
| What else would you like to say about today’s simulated clinical experience? |  |  | 18 |  | 10 |
| Please respond to each question using a five-point scale from Strongly Agree to Strongly Disagree. Choose the answer that you feel best fits your experience. Please indicate the degree to which you currently hold or display each of the beliefs, behaviors and attitudes that are described. Please respond to these questions based on your expectations of each question prior to the Interprofessional Telehealth course. |  |  |  |  |  |
| I feel like I understand when patient safety is at risk and how to escalate care during a telehealth encounter |  | 3 |  | 7 | 1 |
| I feel confident in my physical assessment skills during a telehealth encounter |  | 7 |  | 8 | 5 |
| I feel confident in my ability to elicit a history during a telehealth encounter |  | 4 |  | 9 | 6 |
| I feel confident in my ability to explain my findings and generate a treatment plan during a telehealth encounter |  | 5 |  | 10 | 7 |
| I understand what technology is required and how to guide a patient during a telehealth encounter |  | 1 |  | 6 | 8 |
| I understand how to troubleshoot basic technology failures and optimize settings during a telehealth encounter |  | 8 |  | 5 | 9 |
| I understand how to incorporate new technologies, including patient-owned devices, into a telehalth encounter |  | 9 |  | 4 | 4 |
| I understand the principals of privacy surrounding telehealth encounters |  | 2 |  | 2 | 2 |
| I understand the principals of informed conset for a telehealth encounter |  | 6 |  | 1 | 3 |
| I know how to evaluate federal, state and local facility practice requirements for a telehealth encounter |  | 10 |  | 3 | 10 |
