# Supplementary material for: Development of a Validated Survey to Assess Virtual Interprofessional Education: Cross-Sectional Survey Study With Psychometric Validation
Source: JMIR Form Res. 2026 Oct 7;10:e92933. doi: 10.2196/92933 (PMC13645316; doi:10.2196/92933)
Supplement: Supplementary file 2 [file formative-v10-e92933-s002.docx]

Table 4: Final virtual interprofessional education survey developed through expert review and exploratory psychometric validation in a cross-sectional study of graduate health professions students at Stony Brook University, Stony Brook, New York, USA. The instrument was designed for evaluation of virtual interprofessional education and includes demographic items, telehealth knowledge and skills items, and retained experience and familiarity subscale items derived from survey responses collected across the School of Medicine, School of Nursing, School of Health Professions, and School of Social Work from November 2023 through June 2024.

| **Question Text** | **Response Type / Options** |
| --- | --- |
| Name of Professional Program | ☐ MD ☐ SSW ☐ SHP ☐ SON |
| To which gender do you most closely identify? | ☐ Male ☐ Female ☐ Transgender ☐ Non-binary / non-conforming ☐ Prefer not to say |
| Please indicate your primary ethnicity. | ☐ Not of Hispanic, Latino or Spanish origin ☐ Mexican, Mexican American, Chicano ☐ Puerto Rican ☐ Cuban ☐ Other Hispanic, Latino, or Spanish origin ☐ Prefer not to say |
| Indicate your primary race. | ☐ Black or African American ☐ White ☐ American Indian or Alaskan Native ☐ Chinese ☐ Filipino ☐ Asian Indian ☐ Vietnamese ☐ Korean ☐ Japanese ☐ Samoan ☐ Chamorro ☐ Native Hawaiian/Other Pacific Islander ☐ Other ☐ Prefer not to say |
| Your age | ☐ 21 and below ☐ 22–27 ☐ 28–33 ☐ 34–39 ☐ 40–45 ☐ 46–51 ☐ 52–57 ☐ 58–63 ☐ 64 and up |
| Please indicate the choice below that best describes the amount of experience you have had with using telemedicine/telehealth prior to the course. | ☐ None ☐ Professional occasional use ☐ Professional moderate use ☐ Professional extensive use ☐ Personal use |

| **Q8. Please rate yourself on each of the following knowledge and skills regarding Telehealth using a five-point scale where 5 indicates very familiar and 1 indicates very unfamiliar.** | | | | | |
| --- | --- | --- | --- | --- | --- |
| **Item** | **5 Very Familiar** | **4 Familiar** | **3 Somewhat Familiar** | **2 Unfamiliar** | **1 Very Unfamiliar** |
| Knowledge of Telehealth etiquette | ☐ | ☐ | ☐ | ☐ | ☐ |
| Confidence in using proper patient/provider communication skills in a Telehealth setting | ☐ | ☐ | ☐ | ☐ | ☐ |
| Knowledge of common uses of Telemedicine in my profession | ☐ | ☐ | ☐ | ☐ | ☐ |

| **Q9. Please respond to each question using a five-point scale from Strongly Agree to Strongly Disagree.** | | | | | |
| --- | --- | --- | --- | --- | --- |
| **Item** | **Strongly agree** | **Somewhat agree** | **Neither agree nor disagree** | **Somewhat disagree** | **Strongly disagree** |
| I feel that I can communicate effectively within inter-professional teams | ☐ | ☐ | ☐ | ☐ | ☐ |
| I feel that I can communicate effectively using Telehealth | ☐ | ☐ | ☐ | ☐ | ☐ |
| I have an understanding of how to use Motivational Interviewing to enhance my interview skills with clients/patients | ☐ | ☐ | ☐ | ☐ | ☐ |
| I am familiar with different formalized communication tools utilized within interprofessional Telehealth teams | ☐ | ☐ | ☐ | ☐ | ☐ |
| Working with students from different disciplines enhances my education | ☐ | ☐ | ☐ | ☐ | ☐ |
| My role within an inter-professional team is clearly defined | ☐ | ☐ | ☐ | ☐ | ☐ |
| Health professional students from different disciplines should be educated on establishing virtual collaborative relationships with one another | ☐ | ☐ | ☐ | ☐ | ☐ |
| During their education, health professional students should be involved in teamwork with students from different disciplines in order to understand their respective roles | ☐ | ☐ | ☐ | ☐ | ☐ |
| I will be able to share and exchange ideas in a team discussion | ☐ | ☐ | ☐ | ☐ | ☐ |
| I will feel comfortable in describing my professional role to another team member in a virtual environment | ☐ | ☐ | ☐ | ☐ | ☐ |
| I will gain an enhanced awareness of roles of other professionals on a team in a virtual environment | ☐ | ☐ | ☐ | ☐ | ☐ |
| I will be comfortable engaging in shared decision making with the clients as part of the IP team in a virtual environment | ☐ | ☐ | ☐ | ☐ | ☐ |
| Virtual collaborative learning will help me understand my own professional limitations | ☐ | ☐ | ☐ | ☐ | ☐ |
| Virtual collaborative learning will help me understand the value of other health professionals | ☐ | ☐ | ☐ | ☐ | ☐ |
| Collaborative learning during Telehealth visits is likely to improve services for patients and their households | ☐ | ☐ | ☐ | ☐ | ☐ |
| Learning from students from other professions will make me a more effective member of an Interprofessional team | ☐ | ☐ | ☐ | ☐ | ☐ |
| Working in virtual teams unnecessarily complicates things most of the time | ☐ | ☐ | ☐ | ☐ | ☐ |
| The team approach improves the quality of care to patients | ☐ | ☐ | ☐ | ☐ | ☐ |
| Virtual team meetings foster communication among team members from different disciplines | ☐ | ☐ | ☐ | ☐ | ☐ |
| Patients receiving team care are more likely than other patients to be treated as whole persons | ☐ | ☐ | ☐ | ☐ | ☐ |
| Patients are less satisfied with their care when it is provided by a virtual team | ☐ | ☐ | ☐ | ☐ | ☐ |
| Developing a virtual patient care plan with other team members avoids errors in delivering care | ☐ | ☐ | ☐ | ☐ | ☐ |
| Developing a virtual interdisciplinary patient care plan is excessively time consuming | ☐ | ☐ | ☐ | ☐ | ☐ |
| The give and take among team members help them make better patient care decisions | ☐ | ☐ | ☐ | ☐ | ☐ |
| The virtual team approach makes the delivery of care more efficient | ☐ | ☐ | ☐ | ☐ | ☐ |
| The virtual team approach permits health professionals to meet the needs of family caregivers as well as patients | ☐ | ☐ | ☐ | ☐ | ☐ |
| The team has a good understanding about their respective responsibilities in virtual settings | ☐ | ☐ | ☐ | ☐ | ☐ |
| Team members are usually willing to take into account the convenience of individuals when planning their work | ☐ | ☐ | ☐ | ☐ | ☐ |
| I feel that patient treatment and care are not adequately discussed between and among team members in virtual settings | ☐ | ☐ | ☐ | ☐ | ☐ |
| Individuals on the team share similar ideas about how to treat patients | ☐ | ☐ | ☐ | ☐ | ☐ |
| Team members are willing to discuss individuals’ issues virtually | ☐ | ☐ | ☐ | ☐ | ☐ |
| Team members cooperate with the way care is organized | ☐ | ☐ | ☐ | ☐ | ☐ |
| Team members anticipate when they will need others’ help in virtual settings | ☐ | ☐ | ☐ | ☐ | ☐ |

| **Q10. Please rate your ability to carry out each of the following tasks using a five-point scale where 5 indicates very familiar and 1 indicates very unfamiliar.** | | | | | |
| --- | --- | --- | --- | --- | --- |
| **Item** | **5 Very Familiar** | **4 Familiar** | **3 Somewhat Familiar** | **2 Unfamiliar** | **1 Very Unfamiliar** |
| Function effectively in a virtual interdisciplinary team | ☐ | ☐ | ☐ | ☐ | ☐ |
| Treat team members as colleagues in virtual settings | ☐ | ☐ | ☐ | ☐ | ☐ |
| Identify contributions to patient care that different disciplines can offer | ☐ | ☐ | ☐ | ☐ | ☐ |
| Handle disagreements effectively in virtual settings | ☐ | ☐ | ☐ | ☐ | ☐ |
| Strengthen cooperation among disciplines virtually | ☐ | ☐ | ☐ | ☐ | ☐ |
| Address clinical issues succinctly in virtual interdisciplinary meetings | ☐ | ☐ | ☐ | ☐ | ☐ |
| Develop a virtual interdisciplinary care plan | ☐ | ☐ | ☐ | ☐ | ☐ |
| Recognize when the team is not functioning well in a virtual environment | ☐ | ☐ | ☐ | ☐ | ☐ |
