# Supplementary material for: Development of a Validated Survey to Assess Virtual Interprofessional Education: Cross-Sectional Survey Study With Psychometric Validation
Source: JMIR Form Res. 2026 Oct 7;10:e92933. doi: 10.2196/92933 (PMC13645316; doi:10.2196/92933)
Supplement: Supplementary file 3 [file formative-v10-e92933-s003.docx]

| **Domain** | **Item** | **Final Model Status** | **Source / Development Origin** | **Virtual-Specific Competency?** | **Notes / Rationale** |
| --- | --- | --- | --- | --- | --- |
| Telehealth knowledge and skills | Knowledge of telehealth etiquette | Retained | Newly added / telehealth-specific adaptation | Yes | Captures etiquette and professionalism unique to synchronous telehealth encounters. |
| Telehealth knowledge and skills | Confidence in using proper patient/provider communication skills in a telehealth setting | Retained | Newly added / telehealth-specific adaptation | Yes | Captures communication behaviors required in virtual clinical interactions. |
| Telehealth knowledge and skills | Knowledge of common uses of telemedicine in my profession | Retained | Newly added | Yes | Assesses discipline-specific understanding of telemedicine applications. |
| General interprofessional communication | I feel that I can communicate effectively within interprofessional teams | Removed | Adapted from existing interprofessional communication tools | No | Removed because it reflects general in-person/team communication rather than telehealth-specific competency. |
| Telehealth communication | I feel that I can communicate effectively using telehealth | Retained | Newly added / telehealth-specific adaptation | Yes | Directly assesses confidence communicating through virtual care modalities. |
| Motivational interviewing | I have an understanding of how to use motivational interviewing to enhance my interview skills with clients/patients | Removed | Adapted from existing communication/clinical skills content | No | Removed because it was not specific to interprofessional telehealth and overlapped with broader communication constructs. |
| Interprofessional telehealth communication tools | I am familiar with different formalized communication tools utilized within interprofessional telehealth teams | Removed / candidate item | Newly added / telehealth-specific adaptation | Yes | Telehealth-relevant, but may have been removed if it did not load strongly in the final model. |
| Interprofessional education | Working with students from different disciplines enhances my education | Retained | Adapted from existing interprofessional education tools | No | Captures general IPE attitudes rather than virtual-specific skills. |
| Role clarity | My role within an interprofessional team is clearly defined | Retained | Adapted from existing interprofessional teamwork tools | No | Retained as a core interprofessional competency relevant across care settings. |
| Virtual collaborative relationships | Health professional students from different disciplines should be educated on establishing virtual collaborative relationships with one another | Retained | Newly added / adapted to virtual context | Yes | Captures preparation for collaboration in virtual environments. |
| Interprofessional teamwork education | During their education, health professional students should be involved in teamwork with students from different disciplines in order to understand their respective roles | Retained | Adapted from existing IPE tools | No | Captures foundational IPE exposure and role understanding. |
| Team discussion | I will be able to share and exchange ideas in a team discussion | Retained | Adapted from existing communication/teamwork tools | No | Retained as a general team communication competency. |
| Professional role communication | I will feel comfortable describing my professional role to another team member in a virtual environment | Retained | Adapted from existing role-clarity item with virtual modification | Yes | Distinguishes virtual role communication from in-person team encounters. |
| Role awareness | I will gain an enhanced awareness of roles of other professionals on a team in a virtual environment | Retained | Adapted from existing IPE/role-awareness tools with virtual modification | Yes | Captures virtual interprofessional role recognition. |
| Shared decision-making | I will be comfortable engaging in shared decision making with clients as part of the interprofessional team in a virtual environment | Retained | Adapted from existing collaborative practice tools with virtual modification | Yes | Captures patient/team shared decision-making in telehealth settings. |
| Collaborative learning | Virtual collaborative learning will help me understand my own professional limitations | Retained | Adapted from existing collaborative learning tools with virtual modification | Yes | Virtual-specific wording distinguishes this from traditional IPE items. |
| Collaborative learning | Virtual collaborative learning will help me understand the value of other health professionals | Retained | Adapted from existing collaborative learning tools with virtual modification | Yes | Captures perceived value of interprofessional learning in virtual settings. |
| Telehealth service delivery | Collaborative learning during telehealth visits is likely to improve services for patients and their households | Retained | Newly added / telehealth-specific adaptation | Yes | Captures virtual care delivery and household-centered telehealth context. |
| Interprofessional effectiveness | Learning from students from other professions will make me a more effective member of an interprofessional team | Retained | Adapted from existing IPE tools | No | Measures general perceived benefit of interprofessional learning. |
| Virtual teamwork burden | Working in virtual teams unnecessarily complicates things most of the time | Removed / candidate item | Adapted from ATHCT with virtual modification | Yes | Reverse-valenced item; spreadsheet notes indicate “do not use.” |
| Quality of care | The team approach improves the quality of care to patients | Retained | Adapted from ATHCT | No | Foundational teamwork attitude item retained for comparison with existing in-person tools. |
| Virtual team communication | Virtual team meetings foster communication among team members from different disciplines | Retained / candidate item | Adapted from ATHCT with virtual modification | Yes | Captures communication in virtual team meetings. |
| Whole-person care | Patients receiving team care are more likely than other patients to be treated as whole persons | Retained | Adapted from ATHCT | No | General team-based care attitude item. |
| Virtual team satisfaction | Patients are less satisfied with their care when it is provided by a virtual team | Retained / candidate item | Adapted from ATHCT with virtual modification | Yes | Reverse-valenced item assessing perceived impact of virtual team care. |
| Virtual care planning | Developing a virtual patient care plan with other team members avoids errors in delivering care | Retained | Adapted from ATHCT with virtual modification | Yes | Captures safety and error prevention in virtual interdisciplinary planning. |
| Time burden | Developing a virtual interdisciplinary patient care plan is excessively time consuming | Removed / candidate item | Adapted from ATHCT with virtual modification | Yes | Reverse-valenced item; spreadsheet notes indicate “do not use.” |
| Team decision-making | The give and take among team members helps them make better patient care decisions | Retained | Adapted from ATHCT | No | General team decision-making item. |
| Virtual care efficiency | The virtual team approach makes the delivery of care more efficient | Retained / candidate item | Adapted from ATHCT with virtual modification | Yes | Captures perceived efficiency of virtual team-based care. |
| Family/caregiver-centered care | The virtual team approach permits health professionals to meet the needs of family caregivers as well as patients | Retained | Adapted from ATHCT with virtual modification | Yes | Captures family/caregiver inclusion in virtual care. |
| Virtual interdisciplinary function | Function effectively in a virtual interdisciplinary team | Retained | Adapted from Team Skills Scale with virtual modification | Yes | Core virtual interprofessional teamwork competency. |
| Collegiality | Treat team members as colleagues in virtual settings | Retained | Adapted from Team Skills Scale with virtual modification | Yes | Captures professional respect in virtual collaboration. |
| Discipline contribution | Identify contributions to patient care that different disciplines can offer | Retained | Adapted from Team Skills Scale | No | Core interprofessional role-recognition item. |
| Conflict management | Handle disagreements effectively in virtual settings | Retained | Adapted from Team Skills Scale with virtual modification | Yes | Captures virtual conflict management. |
| Cooperation | Strengthen cooperation among disciplines virtually | Retained | Adapted from Team Skills Scale with virtual modification | Yes | Captures team cooperation in virtual environments. |
| Virtual meeting communication | Address clinical issues succinctly in virtual interdisciplinary meetings | Retained | Adapted from Team Skills Scale with virtual modification | Yes | Captures concise clinical communication in virtual meetings. |
| Virtual care planning | Develop a virtual interdisciplinary care plan | Retained | Adapted from Team Skills Scale with virtual modification | Yes | Captures virtual interprofessional planning competency. |
| Virtual team functioning | Recognize when the team is not functioning well in a virtual environment | Retained | Adapted from Team Skills Scale with virtual modification | Yes | Captures virtual team-process assessment. |
| Team responsibilities | The team has a good understanding about their respective responsibilities in virtual settings | Retained | Adapted from ICS with virtual modification | Yes | Captures shared understanding of roles in virtual settings. |
| Team coordination | Team members are usually willing to take into account the convenience of individuals when planning their work | Retained | Adapted from ICS | No | General team coordination item. |
| Care discussion | I feel that patient treatment and care are not adequately discussed between and among team members in virtual settings | Retained | Adapted from ICS with virtual modification | Yes | Captures perceived communication gaps in virtual team care. |
| Shared treatment approach | Individuals on the team share similar ideas about how to treat patients | Retained | Adapted from ICS | No | General team alignment item. |
| Virtual issue discussion | Team members are willing to discuss individuals’ issues virtually | Retained | Adapted from ICS with virtual modification | Yes | Captures willingness to address care issues in virtual environments. |
| Care organization | Team members cooperate with the way care is organized | Retained | Adapted from ICS | No | General team cooperation item. |
| Anticipating help needs | Team members anticipate when they will need others’ help in virtual settings | Retained | Adapted from ICS with virtual modification | Yes | Captures anticipatory coordination in virtual settings. |
